# Negative life events and internet addiction among college students: role of physical exercise and prosocial behavior

**DOI:** 10.3389/fpsyg.2025.1629818

**Published:** 2025-09-17

**Authors:** Qinyu Huang, Jingjing Li, Jiangyu Wang, Bing Liu

**Affiliations:** ^1^Physical Education College, Shanghai University, Shanghai, China; ^2^Sports Science Research Center, Shanghai University, Shanghai, China

**Keywords:** internet addiction, negative life events, prosocial behavior, physical exercise, college students

## Abstract

**Introduction:**

This study investigates the relationship between negative life events and Internet addiction, as well as the mediating role of prosocial behavior and the moderating role of physical exercise in this process among Chinese college students. Based on the Conservation of Resources Theory and Stress Coping Theory, we constructed a moderated mediation model to analyze the psychological mechanisms underlying students’ Internet addiction.

**Methods:**

A total of 259 undergraduate students from six universities in Shanghai participated in this study. Data were collected through standardized questionnaires and analyzed using moderated mediation analysis.

**Results:**

Prosocial behavior significantly mediates the relationship between negative life events and Internet addiction. Moreover, physical exercise at varying levels exerts differential moderating effects on the relationship between negative life events, prosocial behavior, and Internet addiction.

**Discussion:**

These findings underscore the importance of promoting prosocial behavior and appropriate levels of physical exercise to mitigate the risk of Internet addiction among college populations.

## 1 Introduction

The rapid advancement of the Internet, particularly the rise of short videos, online gaming, and social media, has significantly increased Internet usage among college students. While such frequent use provides convenient access to information and enhances social connectivity, it has also contributed to a growing trend of excessive reliance on the Internet, with some individuals exhibiting signs of Internet addiction. Internet addiction is defined as a condition in which individuals lose control over their Internet usage (Moore et al., 2020), and it has emerged as a significant public health issue of global concern. This addiction can lead to persistent and cumulative negative effects on academic adjustment, mental health, and interpersonal relationships among college students (Baturay and Toker, 2019). Research indicates that the prevalence of Internet addiction among college students in China is as high as 11% (Shao et al., 2018), posing a serious threat to their physical and mental well-being (Shen et al., 2020). Therefore, exploring the influencing factors and underlying mechanisms of Internet addiction among college students holds substantial theoretical significance and offers valuable practical implications for designing psychological interventions and promoting health within higher education contexts.

Existing literature on Internet addiction in college populations has mainly addressed its triggering factors, associated harms, and potential intervention strategies (Baturay and Toker, 2019; Li et al., 2015). Among these factors, negative life events have emerged as significant contributors (Wang et al., 2022; Yan et al., 2014). Negative life events are defined as threatening or stressful situations encountered in daily life (Skaggs and Barron, 2006). Research has shown a significant positive correlation between negative life events and Internet addiction, suggesting that individuals who experience a higher number of negative life events are more likely to engage in excessive and uncontrolled Internet use (Li D. et al., 2016; Li H. et al., 2016). According to the Conservation of Resources Theory, individuals who lack adequate psychological resources, such as social support and self-regulation, are more inclined to adopt avoidance coping strategies when faced with life stressors (Hobfoll and Shirom, 2000). In their quest for immediate gratification and emotional relief, these individuals may resort to excessive Internet use, which can progressively lead to diminished self-control and ultimately result in the development of Internet addiction (Bisen and Deshpande, 2018).

Prosocial behavior, defined by active engagement in forming interpersonal connections, caring for others, and providing assistance (Abdul Kadir, 2024), can significantly support college students in establishing a robust social support network (Man and Jing, 2025). This behavior not only improves the quality of interpersonal relationships and social identity, but also enhances individual’s sense of belonging and self-worth (Klein, 2017). Consequently, it can alleviate the emotional distress associated with negative life events (Ng and Diener, 2022; Raposa et al., 2016). However, the role of prosocial behavior in the relationship between negative life events and Internet addiction among college students remains underexplored.

Existing studies have demonstrated that cognitive behavioral therapy, emotion regulation training, time management and behavioral replacement interventions, family and social support initiatives, as well as healthy lifestyle modifications are crucial strategies for alleviating Internet addiction (Alavi et al., 2021; Liu et al., 2022; Xu et al., 2021). Among these approaches, physical exercise emerges as a beneficial lifestyle choice that not only effectively reduces anxiety, depression, and other negative emotions but also aids college students in coping with stress by enhancing their emotional well-being (Herbert, 2022; Liu et al., 2024). Furthermore, regular participation in physical exercise facilitates social interaction among college students by promoting peer support and communication through team sports and campus activities (Van Luchene and Delens, 2021). Such engagement helps alleviate feelings of loneliness and reduces the likelihood of developing Internet addiction (Wang et al., 2023). Research indicates that adolescents and college students who consistently engage in sports exhibit higher levels of prosocial behavior (Li and Shao, 2022; Luo et al., 2024). Physical exercise enhances self-control and emotional regulation, reduces aggressive and impulsive behaviors, and promotes prosocial behavior (van der Sluys et al., 2024). However, the relationships between physical exercise, prosocial behavior, and the impact of negative life events on Internet addiction among college students warrants further investigation.

The present study aims to elucidate the roles of prosocial behavior and physical exercise in the relationship between negative life events and Internet addiction among college students. This research seeks to inform the development of effective intervention strategies while providing theoretical support for promoting of mental health within this population. Specifically, the study addresses the following questions: (1) What role does prosocial behavior play in the relationship between negative life events and Internet addiction? (2) Does physical exercise moderate the impact of negative life events on both Internet addiction and prosocial behavior?

## 2 Literature review

### 2.1 Negative life events, internet addiction and prosocial behavior

Studies on the causes of Internet addiction indicate that negative life events serve as significant external stressors contributing to its onset and progression (Li D. et al., 2016; Li H. et al., 2016; Tang et al., 2014). Previous studies have shown that negative life events directly predict Internet addiction (Fan et al., 2018). Furthermore, these events can increase both the frequency and duration of Internet use among college students, indirectly contributing to Internet addiction by influencing their emotional states and psychological needs (Baturay and Toker, 2019). According to the Conservation of Resources Theory (Hobfoll, 1989) and the Stress and Coping Theory (Lazarus and Folkman, 1984), individuals experiencing negative life events often suffer losses in core psychological resources, including emotional regulation, cognitive capacity, and social support. The depletion of these resources undermines coping abilities and increases the likelihood of adopting avoidant coping strategies, such as excessive smartphone use, to manage psychological pressure and emotional distress (Bondarchuk et al., 2024). Emotional exhaustion plays a central role in this process. As outlined in Emotional Exhaustion Theory, prolonged exposure to intense negative stimuli can lead to the chronic overconsumption of psychological resources, ultimately resulting in emotional burnout (Maslach et al., 2001). Empirical studies have demonstrated that university students experience significantly heightened emotional exhaustion following academic stress or interpersonal conflict, accompanied by reduced social adaptability and self-regulation, thereby elevating the risk of internet addiction (Wang et al., 2022). The Self-Focus Theory (Mor and Winquist, 2002) posits that negative life events tend to increase individuals’ attention to their internal emotional states. While such self-focused attention may facilitate short-term emotional processing, its prolonged activation can lead to rumination, self-blame, and social withdrawal, ultimately impairing real-world social functioning. Empirical studies have further shown that individuals under heightened stress exhibit significantly elevated levels of self-focused attention, often accompanied by intensified negative emotions such as guilt and sadness (Moberly and Watkins, 2008; Mor et al., 2010). It is evident that the adverse effects of negative life events on college students’ emotional exhaustion, as well as the maladaptive consequences arising from excessive self-focused attention, are well supported by relevant theories and empirical evidence (Ji et al., 2021; Watkins, 2004). Such an internal psychological manifestation, when accumulated to a certain threshold, may further evolve into a tendency to withdraw from social engagement, leading individuals to position themselves as isolated entities and deliberately “conceal” themselves within society through specific means (Chen et al., 2023; Larson and Chastain, 1990). In the contemporary context, the high interactivity and anonymity of online platforms provide college students with an accessible alternative resource for regaining a sense of control and obtaining psychological comfort (Suler, 2004). However, when individuals persist in using the internet as their primary strategy for coping with negative life events, they may, in the short term, alleviate stress by diverting attention (Zhao et al., 2024). Yet, over the long term, this habitual reliance on the internet to regulate negative emotions undermines their capacity for real-world coping and behavioral self-control, thereby impairing the functioning of their self-regulatory system and ultimately predisposing them to internet addiction (Gu et al., 2024; McNicol and Thorsteinsson, 2017). At present, the highly interactive and anonymous nature of online platforms provides a convenient outlet for college students seeking a sense of control and psychological comfort (Lotay, 2016). However, when individuals habitually rely on the Internet as their primary coping mechanism for negative life events, they may only achieve temporary stress relief through distraction (Van Ingen et al., 2016). Over time, this reliance can undermine their capacity for adaptive coping and behavioral regulation, gradually impairing the self-control system and increasing the risk of Internet addiction (LaRose et al., 2003).

In recent years, growing attention has been directed toward the social behavioral traits of individuals, with a particular emphasis on the critical role that prosocial behavior may play in shaping these traits (Esparza-Reig et al., 2021). Prosocial behavior is defined as positive social conduct characterized by helping others, cooperation, and sharing, and serves as an external manifestation of an individual’s social adaptability (Social Learning, 2003). Studies indicate that individuals exhibiting higher levels of prosocial behavior are more likely to receive emotional and resource support through positive social interactions, such as seeking help and engaging in mutual assistance, particularly when confronted with negative life events (Wang et al., 2020). This support effectively mitigates the impact of external pressures (Weinstein and Ryan, 2010). Furthermore, prosocial behavior can indirectly reduce the risk of Internet addiction by enhancing an individual’s self-esteem and self-efficacy (Esparza-Reig et al., 2021; Niemz et al., 2005). Conversely, individuals with lower levels of prosocial behavior are more inclined to seek alternative emotional satisfaction in virtual spaces, which can increase their dependence on the Internet and potentially lead to Internet addiction (Kasap et al., 2023; Kim et al., 2009). Furthermore, social support and prosocial behavior can indirectly mitigate the risk of Internet addiction by enhancing individuals’ emotional resilience and sense of belonging. Students with weak interpersonal ties are more likely to cope with stress through excessive Internet use (Zhang et al., 2018). Moreover, the relationship between stress and addiction is moderated by social adjustment strategies, with a strong sense of belonging acting as a protective factor (Arslan et al., 2021). Existing research suggests that negative life events may exert a suppressive effect on individuals’ prosocial behaviors, particularly in contexts characterized by resource scarcity and stress concentration. Existing research indicates that negative life events may inhibit individuals’ prosocial behaviors, an effect that is particularly pronounced in contexts characterized by resource scarcity and high stress. According to the Conservation of Resources Theory (Hobfoll, 1989) and Self-Determination Theory (Deci and Ryan, 2000), individuals are intrinsically motivated to conserve, protect, and acquire valuable resources, including objective, conditional, energetic, and personal trait resources. When resources are actually lost, face potential loss, or fail to yield the anticipated returns after substantial investment, individuals experience psychological stress (Baumeister et al., 1998). Such stress, in turn, can trigger a range of maladaptive cognitive, emotional, and behavioral responses, ultimately diminishing their willingness to help others or engage in social activities (Li et al., 2022). For example, some Chinese scholars have noted that various forms of competition prevalent in contemporary university campuses, such as major selection or academic awards, are often framed as opportunities for further development. However, when substantial psychological resources are invested without yielding the desired outcomes, the resulting dissonance can lead some students to adopt an extreme interpretation: “If society treats me unfairly, why should I treat society well?.” From this perspective, the essence of stress lies in a “dynamic imbalance of resources.” Under the “cumulative effect of resource loss,” university students’ motivation to actively acquire additional resources may be substantially weakened, prompting them to withdraw from real-world social engagement (Bakker and Mostert, 2024; Liu et al., 2023). In such cases, retreating into the online world to seek alternative resources and a sense of belonging becomes, for many, the preferred course of action (Gu et al., 2024; Mao and Liao, 2025). Thus, in the context of negative life events and Internet addiction, prosocial behavior may serve as a crucial mediating variable (Wang et al., 2022). Specifically, negative life events can lead college students to experience feelings of depression and helplessness, thereby increasing their reliance on the online virtual world for comfort and escape (Fan et al., 2022). However, if individuals can establish and maintain positive relationships and gain social support and acceptance through prosocial behavior, their dependence on Internet may be significantly reduced (Zhao et al., 2020). Therefore, this study proposes *Hypothesis 1*: prosocial behavior mediates the impact of negative life events on Internet addiction among college students.

### 2.2 The moderating effect of physical exercise

Existing research has demonstrated that physical exercise not only contributes directly to the enhancement of individuals’ physical and mental health, but also exerts a significant moderating effect on the relationship between negative life events and psychological outcomes. Empirical studies have shown that physical exercise can serve as a protective buffer in the development of maladaptive behaviors such as Internet addiction and bedtime procrastination triggered by stressful events, negative emotions, or adverse life experiences. For instance, it has been found that among individuals with high levels of physical exercise, the predictive effect of negative life events on addictive behaviors is significantly attenuated (Ji et al., 2024). Additionally, physical exercise has been confirmed to mitigate the adverse effects of stress on adolescents’ psychological stress responses (Wang et al., 2025). Taken together, these findings suggest that physical exercise may play a critical moderating role in the pathway through which negative life events influence internet addiction.

Prosocial behavior is recognized as a significant psychological resource that can mitigate the effects of negative life events on Internet addiction, potentially serve as a mediating factor (Shi et al., 2022). However, previous research indicates that the efficacy of this mediating mechanism may be influenced by individual characteristics (Zheng et al., 2023). As a positive lifestyle, physical exercise plays an important moderating role in psychological regulation, social behavior and addiction prevention (Lin et al., 2020), and may emerge as a vital moderating variable impacting the aforementioned mediation pathways (Yu, 2023). Studies have indicated that individuals engaging in high level of physical exercise are more likely to sustain higher levels of prosocial behavior when confronted with negative life events (Hui et al., 2022). They are also more likely to translate prosocial behavior tendencies into positive actions in real-life scenarios, thereby effectively resisting the onset of Internet addiction (Dou and Shek, 2021). Research has demonstrated a strong correlation between physical exercise and emotional regulation, self-control, as well as the development of social skills (Chen et al., 2022). Through physical exercise, individuals develop an awareness of social norms, empathy, and a sense of responsibility by engaging in rule-following, cooperation, competition, and team communication. These activities closely reflect the fundamental characteristics of prosocial behavior (Zheng et al., 2023). Consequently, physical exercise can indirectly promote prosocial behavior and potentially strengthen its protective effect against Internet addiction. Furthermore, physical exercise enhances individuals’ self-efficacy and psychological resilience, enabling them to maintain prosocial behavior under stress and reduce the likelihood of Internet addiction (Lin et al., 2020).

Therefore, this study proposes *Hypothesis 2*: Physical exercise serves as a moderating factor in both the pathway “negative life events → Internet addiction” and the sequential pathway “negative life events → prosocial behavior → Internet addiction.”

**Figure 1:**
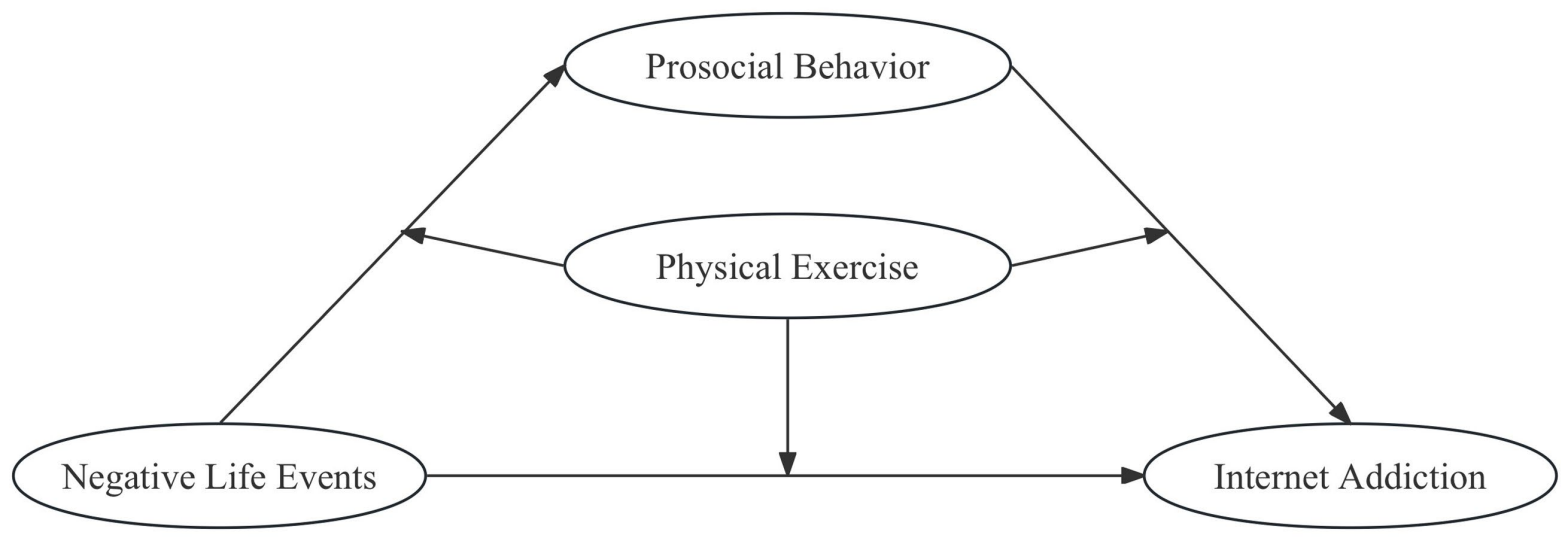
Theoretical model.

This study aims to investigate the mediating role of prosocial behavior in the relationship between negative life events and Internet addiction among college students and analyze the pivotal role of physical exercise. The assumed model is depicted in Figure 1.

## 3 Methods

### 3.1 Participants and procedures

This study received approval from the Ethics Committee of Shanghai University (No. ECSHU 2024–104). The survey targeted students from six universities in Shanghai, including Shanghai University, Shanghai Lixin University of Accounting and Finance, Tongji University, Shanghai Maritime University, University of Shanghai for Science and Technology, and Shanghai University of Medicine & Health Sciences. Using a convenience sampling method, physical education instructors distributed the questionnaire via the “WENJUANXING” online platform during PE classes. Prior to completing the questionnaire, participants were provided with comprehensive information regarding the survey’s purpose, relevant precautions, privacy protection measures, and the handling and storage of the collected data. All participants signed an informed consent form and were informed of their right to withdraw from the study at any time. On average, the questionnaire took approximately 5 min to complete. After excluding questionnaires completed in less than 200 s or those with identical responses for five consecutive questions, a total of 259 valid questionnaires were obtained, comprising 186 male students and 73 female students.

### 3.2 Research tools

#### 3.2.1 The adolescent stressful life events scale

The Adolescent Stressful Life Events Scale (ASLES), developed by Liu et al. (1997), was utilized for measurement (Liu et al., 1997). This scale comprises 27 items (e.g., “Misunderstood or misrepresented,” “Discriminated against”) that assess six dimensions: academic pressure, health adjustment, interpersonal relationships, punishment, loss, and others. Each item is rated on a 5-point Likert scale, scores from 1 to 5 reflect the occurrence of the event and its impact on the individual (1 = “none,” 5 = “extremely severe”). A higher score indicates a greater perceived impact of the event on the individual. In this study, the Cronbach’s *α* coefficient for the Adolescent Stressful Life Events Scale was 0.927, demonstrating good internal consistency.

#### 3.2.2 Prosocial behavior scale

The Prosocial Behavior Scale (PBS), developed by Zhang and Kou (2011), consists of 15 items (e.g., “I like to participate in social welfare activities organized inside and outside the school,” “I am willing to do things for the class community”) encompassing four dimensions: altruism, compliance and public welfare, interpersonal relationships, and prosocial traits (Zhang and Kou, 2011). Items are rated on a 5-point Likert scale, with higher scores indicating greater levels of prosocial behavior. In the present study, the scale demonstrated good internal consistency, with a Cronbach’s *α* coefficient of 0.907.

#### 3.2.3 Physical activity rating scale-3

Physical exercise was measured using the Physical Activity Rating Scale-3 (PARS-3), revised by Liang (1994). This scale assesses physical activity across three dimensions: intensity, duration, and frequency (Liang, 1994). The total physical activity score is calculated using the formula: the mount of physical exercise score = intensity × duration × frequency. Intensity and frequency are rated on a 5-point scale (ranging from 1 to 5), while duration is rated on a 5-point scale from 0 to 4. The total score ranges from 0 to 100, with higher scores indicating greater levels of physical exercise. According to the scale’s classification criteria, scores ≤19 indicate low level of physical exercise, scores between 20 and 42 indicate moderate level of physical exercise, and scores >43 indicate high level of physical exercise. In the present study, the scale demonstrated good internal consistency, with a Cronbach’s α coefficient of 0.805.

#### 3.2.4 Internet addiction scale

The Internet Addiction Scale (IAS), developed by Young (2009), comprises eight items (e.g., “Do you feel preoccupied with the Internet, thinking about previous online activities or anticipating your next online session?”) (Young, 2009). It employs a 5-point scale, where 1 indicates “not at all” and 5 indicates “completely,” with higher scores reflecting greater levels of Internet addiction. The scale demonstrated strong reliability, evidenced by a Cronbach’s α coefficient of 0.859.

### 3.3 Data analysis

SPSS 26.0 software was employed to conduct a common method bias test, as well as to perform statistical descriptions and correlation analysis on the data. Additionally, AMOS was utilized for structural equation modeling to examine the mediating role of prosocial behavior in the relationship between negative life events and Internet addiction among college students. Furthermore, the PROCESS plug-in was applied to investigate the moderating effect of physical exercise.

## 4 Results

### 4.1 Common method bias test

Harman’s single factor test was conducted to assess common method bias. The results indicated that a total of 12 factors with eigenvalues greater than 1 were extracted, with the first factor explaining 26.7% of the variance. This value is below the critical threshold of 40%, suggesting that not serious common method bias is presented in this study.

### 4.2 The descriptive statistics and correlation analysis

The results indicated a significant positive correlation between negative life events experienced by college students and Internet addiction (*r* = 0.524, *p* < 0.01). Furthermore, negative life events were negatively correlated with prosocial behavior (*r* = −0.508, *p* < 0.01). Additionally, prosocial behavior exhibited a significant negative correlation with Internet addiction (*r* = −0.479, *p* < 0.01) (Table 1).

**Table 1:** Descriptive statistics and Pearson’s correlation analysis.

Variables	*M* ± SD	1	2	3	4
1. Negative life events	1.19 ± 0.71	1			
2. Prosocial behavior	3.65 ± 0.86	−0.508**	1		
3. Physical exercise	19.98 ± 23.45	0.013	0.220**	1	
4. Internet addiction	1.88 ± 0.67	0.524**	−0.479**	0.004	1

M, Mean; SD, Standard Deviation. **p < 0.01.

### 4.3 The mediating effect of prosocial behavior

The fitting indices for the model examining the effects of negative life events and prosocial behavior on Internet addiction among college students are as follows: *χ*^2^/*df* = 1.372, RMR = 0.036, GFI = 0.962, AGFI = 0.941, NFI = 0.939, TLI = 0.977, CFI = 0.983, and RMSEA = 0.038, indicating a good model fit (Table 2). The results from the structural equation modeling reveal that negative life events significantly and positively predict both Internet addiction (*β* = 0.345, *p* < 0.001) and prosocial behavior (β = −0.646, *p* < 0.001) among college students. Additionally, prosocial behavior has a significant negative predictive effect on Internet addiction (β = −0.335, *p* < 0.001) (Table 3). Therefore, *Hypothesis 1* is supported.

**Table 2:** Model fit indices.

Fitness index	*χ*^2^/*df*	RMR	GFI	AGFI	NFI	TLI	CFI	RMSEA
Value	1.372	0.036	0.962	0.941	0.939	0.977	0.983	0.038

**Table 3:** Path coefficient of negative life events on Internet addiction.

Path	*β*	*S.E.*	*C.R.*	*p*
Negative Life Events → Internet Addiction	0.345	0.106	3.88	***
Negative Life Events → Prosocial Behavior	−0.646	0.149	−6.609	***
Prosocial Behavior → Internet Addiction	−0.335	0.07	−3.755	***

***p < 0.001.

### 4.4 The moderating effect of physical exercise

This study examines the relationship between negative life events (independent variable) and Internet addiction (dependent variable) among college students, with prosocial behavior serving as a mediating variable and physical exercise as a moderating variable. Utilizing the PROCESS plug-in v4.2 for SPSS 26.0 software, we selected Model 59 to investigate the moderating effect of physical exercise.

The results indicated that the interaction between negative life events and physical exercise exerted a significant positive predictive effect on the prosocial behavior of college students (*β* = 0.212, *p* < 0.01) (Table 4). This suggests that physical exercise negatively moderates the relationship between negative life events and prosocial behavior among college students. Specifically, at low level of physical exercise, the effect size of negative life events on prosocial behavior was −0.637 [95% CI (−0.758, −0.516)], whereas at high level of physical exercise, the effect size was −0.245 [95% CI (−0.420, −0.070)] (Table 5). This indicates that physical exercise can attenuate the detrimental impact of negative life events on prosocial behavior.

**Table 4:** Regression analysis of variable relationships in the model.

Result variable	Predictive variable	*R*	*R* ^2^	*F*	*β*	*t*
Prosocial behavior	Negative life events	0.586	0.344	44.473***	−0.457***	−8.634
	Physical exercise				0.233***	4.585
	Negative life events * physical exercise				0.212**	3.652
Internet addiction	Negative life events	0.612	0.374	30.278***	0.330***	5.563
	Prosocial behavior				−0.357***	−4.020
	Physical exercise				0.105	1.662
	Negative life events * physical exercise				−0.230**	−3.738
	Prosocial behavior * physical exercise				−0.167	−1.515

*p < 0.05, **p < 0.01, ***p < 0.001.

**Table 5:** The relationship between negative life events and prosocial behaviors in college students with different levels of physical exercise.

Group	Effect value	SE	*t*	Bootstrap 95% CI	
				Boot LLCI	Boot ULCI
Low level of physical exercise	−0.637	0.061	−10.374	−0.758	−0.516
Moderate level of physical exercise	−0.457	0.053	−8.634	−0.561	−0.352
High level of physical exercise	−0.245	0.089	−2.753	−0.420	−0.070

Furthermore, the interaction between negative life events and physical exercise significantly predicted Internet addiction (*β* = −0.23, *p* < 0.01), indicating that physical exercise negatively moderates the relationship between negative life events and Internet addiction among college students. At low level of physical exercise, the effect of negative life events on Internet addiction was 0.526 [95% CI (0.383, 0.669)], while at high level of physical exercise, the impact of negative life events on Internet addiction was not significant [95% CI (−0.082, 0.281)] (Table 6). This indicates that physical exercise can buffer the impact of negative life events on Internet addiction.

**Table 6:** The relationship between negative life events and Internet addiction among college students with different levels of physical exercise.

Group	Effect value	SE	*t*	Bootstrap 95% CI	
				Boot LLCI	Boot ULCI
Low level of physical exercise	0.526	0.073	7.256	0.383	0.669
Moderate level of physical exercise	0.330	0.059	5.563	0.213	0.447
High level of physical exercise	0.100	0.092	1.081	−0.082	0.281

**Figure 2:**
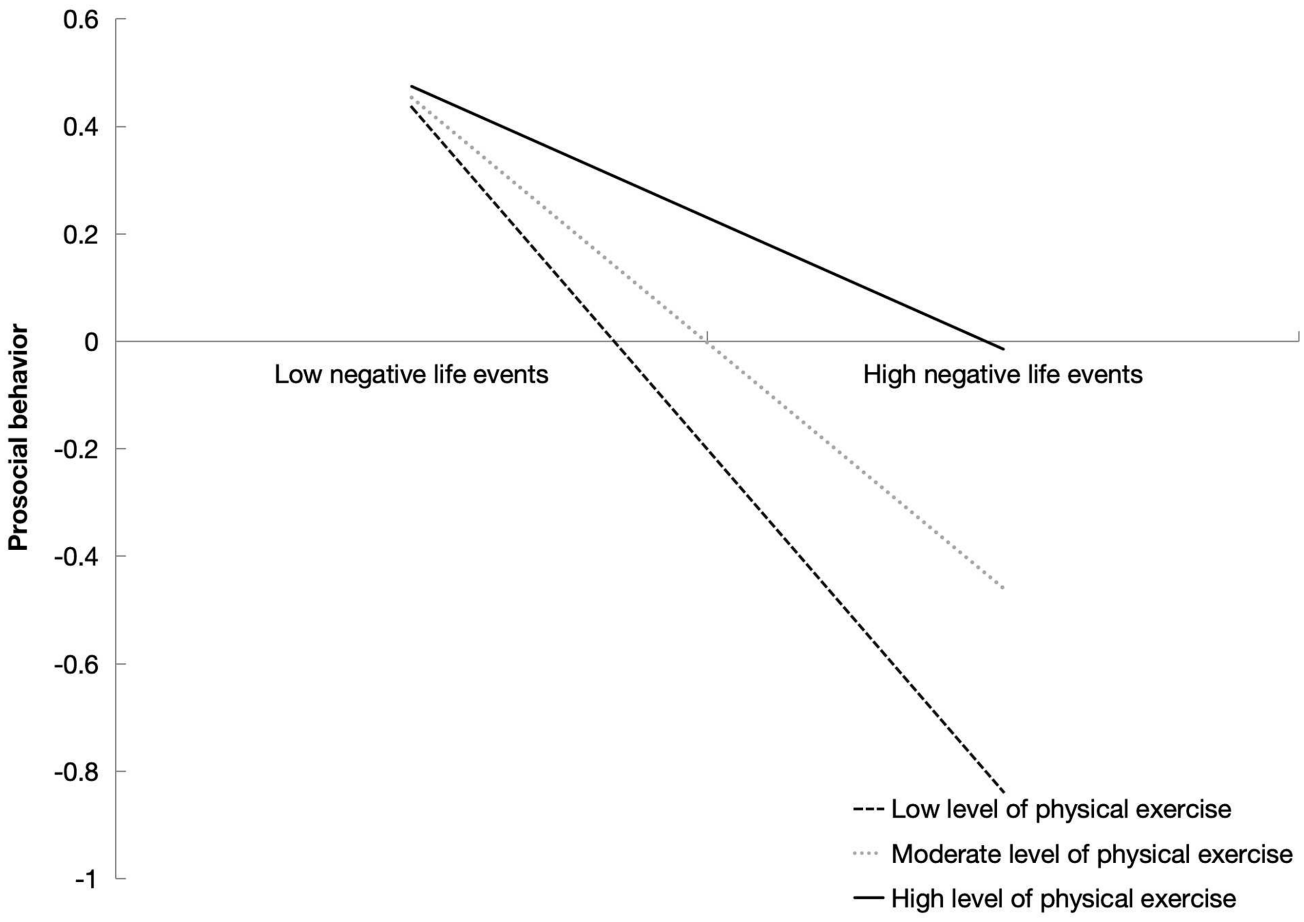
The relationship between negative life events and prosocial behavior in college students with different levels of physical exercise.

**Figure 3:**
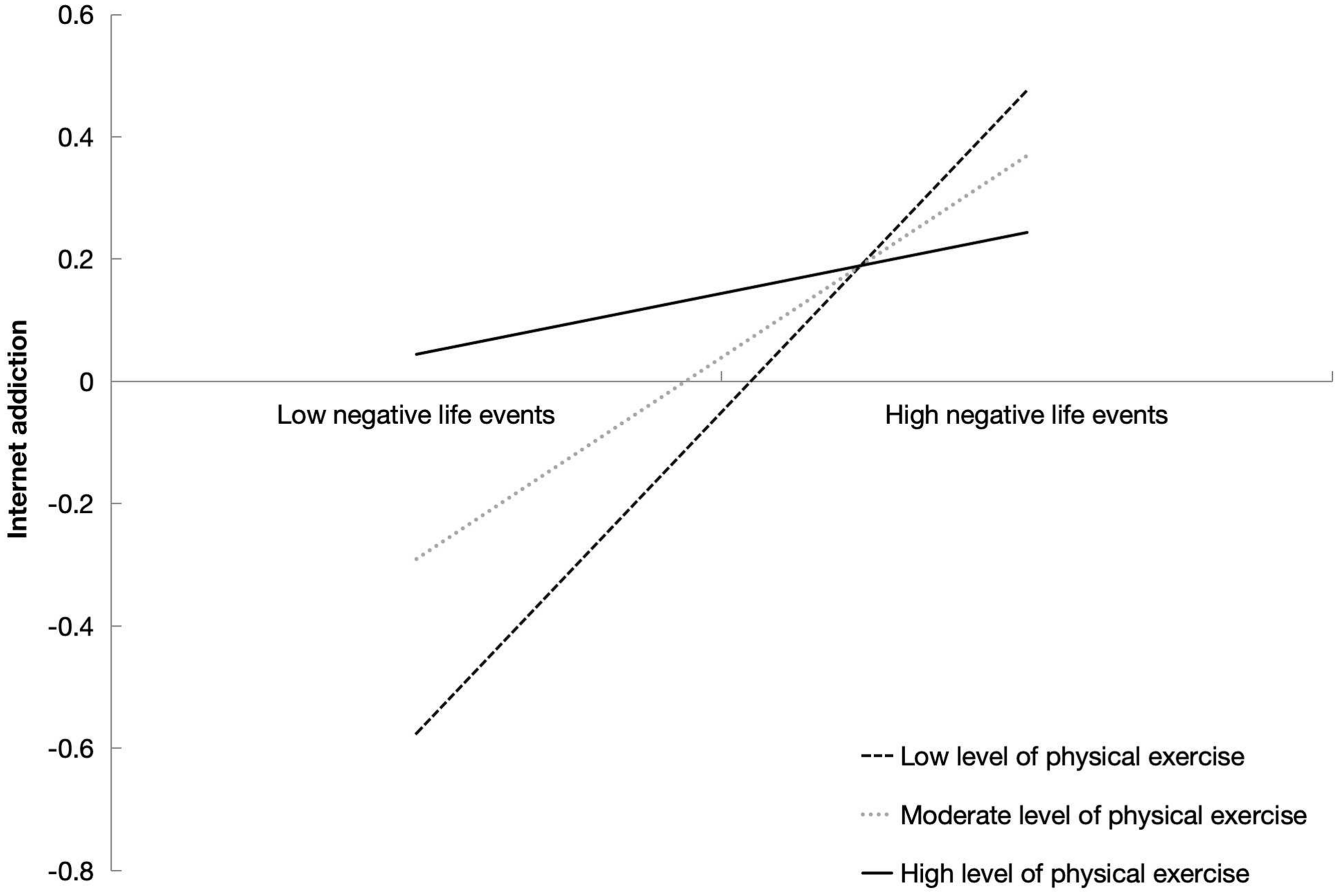
The relationship between negative life events and Internet addiction under different levels of physical exercise among college students.

**Figure 4:**
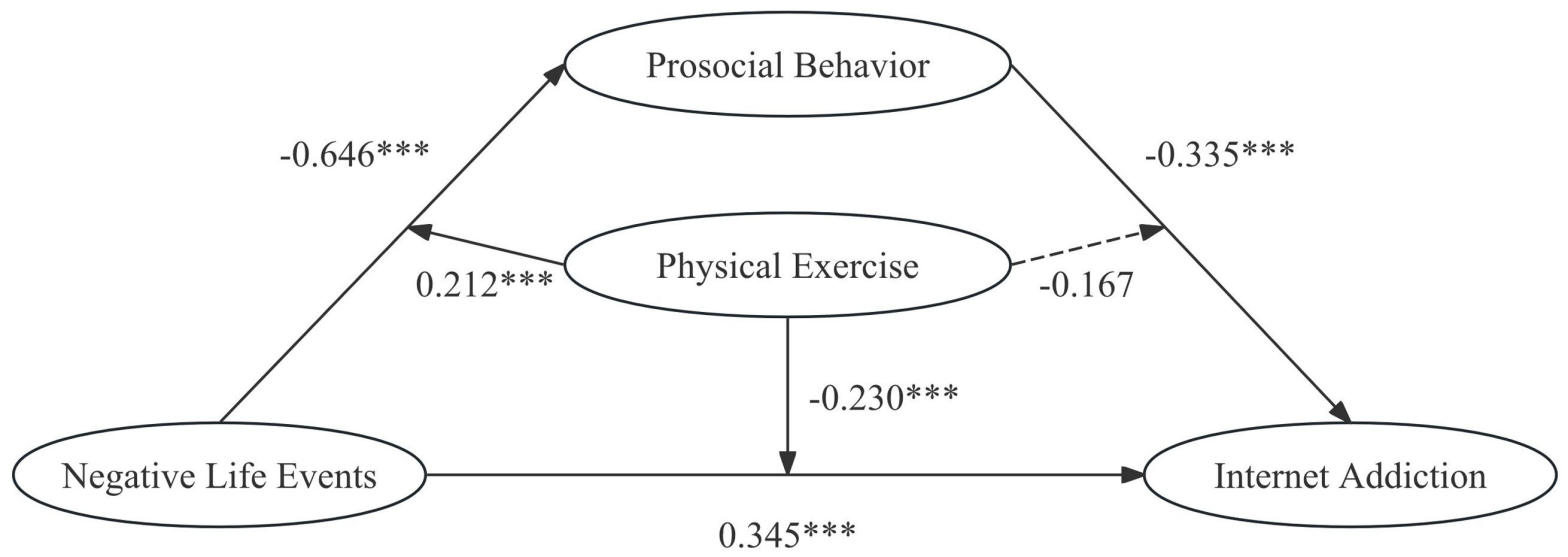
A moderated mediation model illustrating the effect of negative life events on Internet addiction among college students. ***p < 0.001.

Additionally, the interaction between prosocial behavior and physical exercise (*β* = −0.167, *p* > 0.05) did not significantly affect Internet addiction, suggesting that the level of physical exercise does not moderate the relationship between prosocial behavior and Internet addiction. Overall, physical exercise exerts a buffering effect on both pathways through which negative life events influence Internet addiction, but it shows no significant moderating effect on the relationship between prosocial behavior and Internet addiction (Figures 2–4). Then, *Hypothesis 2* was partially supported.

## 5 Discussion

The research findings indicate that prosocial behavior mediates the relationship between negative life events and Internet addiction among college students. Additionally, physical exercise serves as a moderator in this mediating process (Figures 3, 4), demonstrating a differentiated moderating effect based on varying levels of physical exercise. Specifically, at low level of physical exercise, physical exercise moderates the impact of negative life events on prosocial behavior, as well as the Internet addiction among college students. Conversely, at high level of physical exercise, physical exercise continues to moderate the effect of negative life events on prosocial behavior, its moderating influence on the relationship between negative life events and Internet addiction becomes insignificant. These results elucidate the complex psychological mechanisms underlying individuals’ stress coping strategies and Internet addiction, providing a theoretical foundation for developing physical exercise intervention strategies.

### 5.1 The mediating role of prosocial behavior in the impact of negative life events on internet addiction among college students

The findings of this study reveal a significant positive correlation between negative life events and Internet addiction, which aligns with prior research (Wang et al., 2022; Zeng P. et al., 2023; Zeng Z. et al., 2023). Furthermore, the study highlights that negative life events can trigger Internet addiction by increasing psychological stress and negative emotions (Ji et al., 2025; Li D. et al., 2016; Li H. et al., 2016). When faced with academic pressure, interpersonal conflicts, or family disputes, many college students choose to alleviate their emotional distress through online activities such as gaming and social media use (Li D. et al., 2016; Li H. et al., 2016). However, the duration of this effect tends to be relatively short-term. Over time, it may exacerbate reliance on the Internet, ultimately leading to the development of Internet addiction (Li et al., 2015).

According to the Stress and Coping Theory, college students often experience heightened psychological stress when faced with negative life events, which predisposes them to negative emotions such as anxiety and depression (Sun et al., 2017). As a result, they may resort to avoidance coping strategies to alleviate uncomfortable experiences (Chou et al., 2015). Among these strategies, Internet addiction stands out as a prevalent avoidant behavior, fundamentally functioning as a maladaptive coping mechanism (Li et al., 2019). In contrast, prosocial behavior represents a positive form of social adaptation, providing effective emotional buffering during stressful situations (Xue et al., 2022). By participating in volunteer services, club activities, or engaging in altruistic actions in their daily lives, college students not only gain positive emotional experiences but also cultivate stronger social connections through interactions with others (Jiang et al., 2021). This engagement enables them to receive emotional support and a sense of belonging from external sources, thereby alleviating feelings of loneliness and helplessness (Oyedokun et al., 2023) and reducing their dependence on the online virtual environment (Dou and Shek, 2021). Research indicates that individuals who exhibit higher levels of prosocial behavior are more likely to seek real-world support and actively confront stress (Xu et al., 2025), thereby diminishing their vulnerability to Internet addiction.

From a theoretical perspective, positive psychology suggests that prosocial behavior enhances an individual’s positive emotions, psychological resilience, and subjective well-being, thereby mitigating maladaptive behavioral responses induced by stress (Towsyfyan et al., 2021). Furthermore, Social Connection Theory posits that the closer an individual’s social ties, the more their behavior is constrained by social norms, which reduces the likelihood of engaging in problematic behaviors (Umberson et al., 2010; Valente, 2010). Prosocial behavior not only reinforces social connections but also enhances self-control and value recognition, thereby further diminishing reliance on addictive behaviors associated with the Internet (Zeng P. et al., 2023; Zeng Z. et al., 2023). Thus, prosocial behavior acts as a key mediator between negative life events and Internet addiction. It mitigates the harmful impact of stress by providing positive social resources and psychological support. Moreover, by enhancing social adaptability, prosocial behavior facilitates the development of more constructive coping strategies among college students, thereby reducing the likelihood that negative life events will lead to Internet addiction.

In China, prosocial behavior encompasses helping others, cooperation, and sharing, deeply rooted in Confucian values such as benevolence and courtesy. These cultural tenets emphasize individual responsibilities and obligations toward families, collectives, and society (Zhou et al., 2021). This value system fosters strong group identity and collective consciousness among college students, facilitating the acquisition of social support and recognition through prosocial actions. Such mechanisms aid in alleviating psychological stress caused by negative life events (Man and Jing, 2025). Empirical studies corroborate this perspective. For instance, some investigations have demonstrated that higher levels of prosocial behavior in college students correlate with improved psychological adaptability and lower levels of loneliness and anxiety (Zhou, 2025). Other studies have also indirectly highlighted that prosocial behavior may mediate the relationship between life stress and Internet addiction among Chinese adolescents. Specifically, increased stress is associated with lower levels of prosocial behavior and a greater tendency towards Internet addiction (Yang et al., 2017). These findings further reinforce the mediating role of prosocial behavior in the relationship between negative life events and Internet addiction.

It is worth noting that although prosocial behavior is often regarded as a relatively stable individual trait, its actual expression is highly susceptible to contextual influences. Empirical studies have shown that under acute stress, individuals’ cognitive and emotional resources are easily depleted, thereby inhibiting the overt display of prosocial behavior (Forbes et al., 2024; Nitschke et al., 2022). According to the Trait Activation Theory, the expression of traits is contingent upon external situational cues, and adverse environments may even suppress such expression (Prentice et al., 2019). In the context of negative life events, individuals may possess a predisposition toward prosocial behavior, yet their behavioral expression may be attenuated by the stressful environment, thereby limiting opportunities to achieve emotional regulation through social connectedness. The proposed mediating pathway “negative life events → prosocial behavior → Internet addiction” though spanning different temporal dimensions, is theoretically coherent. Specifically, the expression of prosocial behavior may be hindered under stress, weakening its regulatory function and consequently increasing the risk of individuals relying on internet use as an alternative means of psychological gratification.

### 5.2 The moderating effect of physical exercise

The findings of this study revealed that physical exercise exerted significant moderating effects on both the “negative life events → prosocial behavior” and “negative life events → Internet addiction” pathways, whereas its moderating effect on the “prosocial behavior → Internet addiction” pathway was not statistically significant. This suggests that the protective role of physical exercise may primarily manifest during the stages of stress response and psychological resource regulation.

Two potential mechanisms have been supported by prior research. On one hand, physical exercise effectively reduces perceived stress and negative emotional responses, thereby mitigating the adverse impacts of negative life events. For example, research has found that during major examinations in Chinese universities, such as final exams, postgraduate entrance examinations, and the College English Test (CET) 4 and 6, students who regularly participate in physical activities are able to significantly buffer the negative emotional effects of stress, with moderate- and low-intensity exercise showing particularly pronounced benefits (Zhang et al., 2023). Another study on adolescent internet addiction demonstrated that physical exercise moderated the relationship between stress and problematic internet use, lending empirical support to the “stress-buffering model” (Brailovskaia et al., 2018; Lin et al., 2020). On the other hand, physical exercise may also stimulate individuals’ social motivation, psychological resilience, and sense of social connectedness, enabling them to maintain the capacity and willingness to engage in prosocial behaviors even under stress, thereby reducing reliance on the internet as an avoidant coping strategy. Empirical evidence suggests that physical activity alleviates loneliness, enhances social belonging, and promotes real-world social interactions (Wan et al., 2021). Additionally, by improving self-esteem and subjective well-being, physical exercise may indirectly foster prosocial behavior among university students, subsequently lowering the risk of internet addiction (Zhu et al., 2025). Thus, physical exercise may not only exert a “stress-buffering” effect through emotional regulation but also indirectly enhance the expression of prosocial behaviors by strengthening psychological resources such as social motivation, belongingness, and self-efficacy, thereby offering effective protection against internet addiction (Jiang and Bian, 2025; Xu and Tang, 2024; Zhu et al., 2025).

The results of this study also indicate that low level of physical exercise could enhance the prosocial behavior and effectively mitigate Internet addiction among college students. High level of physical exercise buffers the effects of negative life events on prosocial behavior, However, its moderating effect on reducing Internet addiction becomes insignificant. This finding aligns with the inverted U hypothesis of physical exercise intervention, which posits that moderate physical exercise is most beneficial for psychological health, whereas excessive physical exercise may lead to physiological fatigue and psychological strain, thereby diminishing its positive regulatory effects (McMorris et al., 2015). Empirical studies have demonstrated that moderate participation in physical exercise, as a significant means of enhancing collective identity, fosters interpersonal interaction and a sense of belonging within the group, thereby promoting prosocial behavior (Wan et al., 2021). Additionally, research has shown that moderate level of physical exercise significantly enhance the prosocial behavior and psychological resilience of college students; conversely, excessive physical exercise (e.g., more than 2 h of high-intensity exercise per day) can reduce social participation (Moore et al., 2020). It has also been suggested that physical exercise indirectly mitigates Internet addiction by enhancing positive emotions (Cheng et al., 2023), but few scholars have distinguished the relationship between the levels of physical exercise and internet addiction. Furthermore, some studies indicate that certain students in the high-exercise group display a degree of exercise dependence and social avoidance (Hausenblas and Downs, 2002).

From the perspective of Conservation of Resources Theory, physical exercise acts as a means of supplementing psychological resources, thereby enhancing an individual’s positive emotions and coping abilities (Pretty et al., 2003). This enhancement fosters greater psychological resilience in the face of life stress. However, when the levels of physical exercise exceeds an individual’s tolerance or regulatory range, the benefits derived from these resources may begin to diminish, or even lead to new resource depletion (Meyler et al., 2023). For instance, prolonged high-intensity exercise can undermine the protective effects of prosocial behavior by limiting an individual’s time and energy for social interactions and reducing engagement in real-world social situations (Wiersma, 2000). Additionally, physical exercise motivated by external evaluations may not enhance psychological resources; instead, it may increase anxiety and social avoidance, thus weakening its moderating role in the impact of negative life events on Internet addiction (Xiang et al., 2024).

## 6 Limitations

Despite yielding several important findings, this study is subject to certain limitations. First, the use of a cross-sectional design, by its very nature, lacks a temporal dimension, which constrains causal inference and limits the ability to capture dynamic changes. Such a design is inherently static, making it difficult to control for confounding factors and to fully exclude the influence of individual characteristics. In the current context, where negative life events increasingly drive college students to “withdraw” from society through Internet addiction, future research should aim to minimize the impact of confounding variables by ensuring balanced sample distribution across age, gender, year of study, academic major, and personality traits. Furthermore, stratifying participants according to confounder levels and incorporating these variables into statistical models, such as through logistic regression, would allow for adjusted analyses of the associations between prosocial behavior, physical exercise, and Internet addiction among college students. Such methodological refinements would enhance the rigor and scientific validity of the findings.

## 7 Conclusion

This study demonstrates that prosocial behavior mediates the effect of negative life events on college students’ Internet addiction through the establishment of a mediated model. Furthermore, physical exercise acts as a partial moderator within this model, particularly at varying levels of physical exercise. These findings elucidate the mechanisms by which negative life events, prosocial behavior, and physical exercise influence Internet addiction, thereby providing a theoretical foundation and strategic approach for mental health education and intervention among college students.

## Data Availability

The raw data supporting the conclusions of this article will be made available by the authors, without undue reservation.
